# 4,4′-[Propane-1,2-diyl­bis(nitrilo­methyl­idyne)]­dibenzo­nitrile–4,4′-[ethane-1,2-diylbis(nitrilo­methyl­idyne)]dibenzonitrile [0.796 (2)/0.204 (2)]

**DOI:** 10.1107/S1600536808031097

**Published:** 2008-10-18

**Authors:** Hoong-Kun Fun, Reza Kia, Hadi Kargar

**Affiliations:** a X-ray Crystallography Unit, School of Physics, Universiti Sains Malaysia, 11800 USM, Penang, Malaysia; b Department of Chemistry, School of Science, Payame Noor University (PNU), Ardakan, Yazd, Iran

## Abstract

The title cocrystal, 0.796C_19_H_16_N_4_·0.204C_18_H_14_N_4_, is a disordered mixture of two potentially bidentate Schiff base ligands. The difference in the two components of the cocrystal is the replacement of the methyl group in the linkage between the imine N atoms in the major component of the Schiff base ligand by an H atom. The imino (C=N) functional groups are coplanar with the benzene rings (only the major component)  and extend in opposite directions (both components). Inter­molecular π–π inter­actions with a centroid-to-centroid distance of 3.7371 (8) Å are observed in the crystal packing.

## Related literature

For values of bond lengths, see: Allen *et al.* (1987 ▶). For related structures, see, for example: Li *et al.* (2005 ▶); Bomfim *et al.* (2005 ▶); Glidewell *et al.* (2005 ▶, 2006 ▶); Sun *et al.* (2004 ▶); Fun, Kia & Kargar (2008 ▶); Fun, Kargar & Kia (2008 ▶).
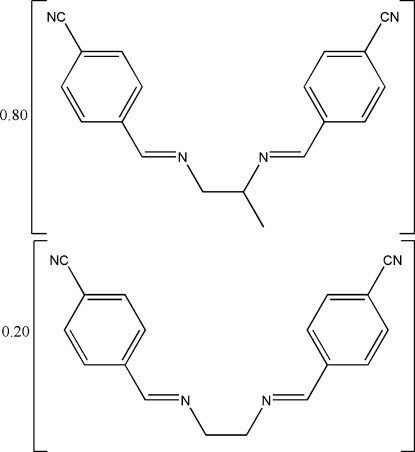


## Experimental

### 

#### Crystal data

0.796C_19_H_16_N_4_·0.204C_18_H_14_N_4_
*M* *_r_* = 297.68
Triclinic, 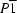

*a* = 9.2370 (2) Å
*b* = 9.7769 (2) Å
*c* = 9.8169 (2) Å
α = 81.846 (1)°
β = 75.098 (1)°
γ = 67.421 (1)°
*V* = 790.15 (3) Å^3^
*Z* = 2
Mo *K*α radiation
μ = 0.08 mm^−1^
*T* = 100.0 (1) K
0.39 × 0.38 × 0.27 mm

#### Data collection

Bruker SMART APEXII CCD area-detector diffractometer
Absorption correction: multi-scan (**SADABS**; Bruker, 2005 ▶) *T*_min_ = 0.890, *T*_max_ = 0.979
17295 measured reflections
4628 independent reflections
3746 reflections with *I* > 2σ(*I*)
*R*_int_ = 0.020

#### Refinement

*R*[*F* ^2^ > 2σ(*F* ^2^)] = 0.059
*wR*(*F* ^2^) = 0.182
*S* = 1.04
4628 reflections
230 parameters
H-atom parameters constrained
Δρ_max_ = 0.57 e Å^−3^
Δρ_min_ = −0.48 e Å^−3^

### 

Data collection: *APEX2* (Bruker, 2005 ▶); cell refinement: *SAINT* (Bruker, 2005 ▶); data reduction: *SAINT*; program(s) used to solve structure: *SHELXTL* (Sheldrick, 2008 ▶); program(s) used to refine structure: *SHELXTL*; molecular graphics: *SHELXTL*; software used to prepare material for publication: *SHELXTL* and *PLATON* (Spek, 2003 ▶).

## Supplementary Material

Crystal structure: contains datablocks global, I. DOI: 10.1107/S1600536808031097/ez2140sup1.cif

Structure factors: contains datablocks I. DOI: 10.1107/S1600536808031097/ez2140Isup2.hkl

Additional supplementary materials:  crystallographic information; 3D view; checkCIF report

## Figures and Tables

**Fig. 1.**
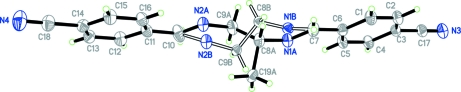
The molecular structure of the title cocrystal, with atom labels and 50% probability ellipsoids for non-H atoms. Open bonds indicate the minor component.

**Fig. 2.**
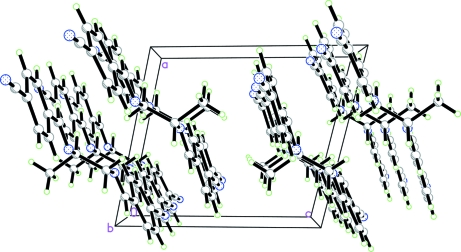
The crystal packing of the major component of (I), viewed down the b-axis.

## supplementary crystallographic information

### Comment

Schiff bases are among the most prevalent mixed-donor ligands in the field of coordination chemistry. They play an important role in the development of catalysts and enzymatic reactions, magnetic materials and supramolecular architectures. Structures of Schiff bases derived from substituted benzaldehydes and closely related to the title compound have been reported recently (Li *et al.*, 2005; Bomfim *et al.*, 2005; Glidewell *et al.*, 2005, 2006; Sun *et al.*, 2004; Fun, Kargar & Kia, 2008; Fun, Kia & Kargar, 2008).

The title cocrystal consists of two bidentate Schiff base ligands. The bond lengths and angles in the two molecules (I, Fig. 1) are within normal ranges (Allen *et al.*, 1987). In the major component, the imino (C=N) functional groups are coplanar with the benzene rings. The difference in the two components of the co-crystal, which are disordered over two sites, is the replacement of the methyl group in the major component by a hydrogen atom in the linkage between the imino N atoms. The ratio of the refined site occupancy factors of the major and minor components is 0.796 (2)/0.204 (2). The short distance between the centroids of the six-membered rings indicates the existence of π-π interactions (Fig. 2), with a *Cg*1···*Cg*1 distance of 3.7371 (8) Å (symmetry code: -*x*, -*y*, 2 - *z*; *Cg*1 is the centroid of the C11–C16 benzene ring).

### Experimental

The synthetic method has been described previously (Fun, Kargar & Kia, 2008). Single crystals suitable for *X*-ray diffraction were obtained by evaporation of an ethanol solution at room temperature.

### Refinement

H atoms were positioned geometrically and refined using a riding model with C—H = 0.93–0.98 Å. The *U*_iso_ values were constrained to be 1.5*U*_eq_ of the carrier atom for the methyl H atoms and 1.2*U*_eq_ for the remaining H atoms. A rotating-group model was used for the methyl groups. Incorrect bond lengths of the methylene bridge, plus the presence of large peaks in the difference Fourier map near to the methylene bridge, led us to suspect positional disorder of this segment of the title compound. The refined ratio of the site-occupany factors for the disorder parts is 0.796 (2)/0.204 (2).

### Crystal data

**Table:**

0.796C_19_H_16_N_4_·0.204C_18_H_14_N_4_	*Z* = 2
*M**_r_* = 297.68	*F*(000) = 313
Triclinic, *P*1	*D*_x_ = 1.251 Mg m^−^^3^
Hall symbol: -P 1	Mo *K*α radiation, λ = 0.71073 Å
*a* = 9.2370 (2) Å	Cell parameters from 6569 reflections
*b* = 9.7769 (2) Å	θ = 3.2–30.8°
*c* = 9.8169 (2) Å	µ = 0.08 mm^−^^1^
α = 81.846 (1)°	*T* = 100 K
β = 75.098 (1)°	Block, colourless
γ = 67.421 (1)°	0.39 × 0.38 × 0.27 mm
*V* = 790.15 (3) Å^3^	

### Data collection

**Table:**

Bruker SMART APEXII CCD area-detector diffractometer	4628 independent reflections
Radiation source: fine-focus sealed tube	3746 reflections with *I* > 2σ(*I*)
graphite	*R*_int_ = 0.020
φ and ω scans	θ_max_ = 30.1°, θ_min_ = 2.2°
Absorption correction: multi-scan (*SADABS*; Bruker, 2005)	*h* = −13→13
*T*_min_ = 0.890, *T*_max_ = 0.979	*k* = −13→13
17295 measured reflections	*l* = −13→13

### Refinement

**Table:**

Refinement on *F*^2^	Primary atom site location: structure-invariant direct methods
Least-squares matrix: full	Secondary atom site location: difference Fourier map
*R*[*F*^2^ > 2σ(*F*^2^)] = 0.059	Hydrogen site location: inferred from neighbouring sites
*wR*(*F*^2^) = 0.182	H-atom parameters constrained
*S* = 1.05	*w* = 1/[σ^2^(*F*_o_^2^) + (0.0996*P*)^2^ + 0.2311*P*] where *P* = (*F*_o_^2^ + 2*F*_c_^2^)/3
4628 reflections	(Δ/σ)_max_ < 0.001
230 parameters	Δρ_max_ = 0.57 e Å^−^^3^
0 restraints	Δρ_min_ = −0.48 e Å^−^^3^

### Special details

**Table:**

Experimental. The low-temperature data was collected with the Oxford Cyrosystem Cobra low-temperature attachment.
Geometry. All e.s.d.'s (except the e.s.d. in the dihedral angle between two l.s. planes) are estimated using the full covariance matrix. The cell e.s.d.'s are taken into account individually in the estimation of e.s.d.'s in distances, angles and torsion angles; correlations between e.s.d.'s in cell parameters are only used when they are defined by crystal symmetry. An approximate (isotropic) treatment of cell e.s.d.'s is used for estimating e.s.d.'s involving l.s. planes.
Refinement. Refinement of *F*^2^ against ALL reflections. The weighted *R*-factor *wR* and goodness of fit *S* are based on *F*^2^, conventional *R*-factors *R* are based on *F*, with *F* set to zero for negative *F*^2^. The threshold expression of *F*^2^ > σ(*F*^2^) is used only for calculating *R*-factors(gt) *etc*. and is not relevant to the choice of reflections for refinement. *R*-factors based on *F*^2^ are statistically about twice as large as those based on *F*, and *R*- factors based on ALL data will be even larger.

### Fractional atomic coordinates and isotropic or equivalent isotropic displacement parameters (Å^2^)

**Table:**

	*x*	*y*	*z*	*U*_iso_*/*U*_eq_	Occ. (<1)
N1A	0.44472 (19)	0.58449 (16)	0.76563 (18)	0.0277 (3)	0.796 (2)
N2A	0.31537 (17)	0.28586 (16)	0.96983 (15)	0.0294 (3)	0.796 (2)
C8A	0.41965 (19)	0.44248 (17)	0.78683 (17)	0.0276 (3)	0.796 (2)
H8AA	0.5235	0.3602	0.7675	0.033*	0.796 (2)
C9A	0.3328 (2)	0.42957 (19)	0.93985 (17)	0.0311 (4)	0.796 (2)
H9AA	0.3933	0.4409	1.0021	0.037*	0.796 (2)
H9AB	0.2275	0.5079	0.9569	0.037*	0.796 (2)
C19A	0.3162 (2)	0.44326 (18)	0.68371 (15)	0.0302 (4)	0.796 (2)
H19A	0.3770	0.4426	0.5883	0.045*	0.796 (2)
H19B	0.2204	0.5308	0.6954	0.045*	0.796 (2)
H19C	0.2873	0.3569	0.7039	0.045*	0.796 (2)
N1B	0.4751 (7)	0.5817 (7)	0.8069 (6)	0.0236 (13)*	0.204 (2)
N2B	0.2894 (7)	0.2948 (6)	0.8959 (6)	0.0294 (3)	0.204 (2)
C8B	0.4619 (7)	0.4375 (6)	0.8536 (6)	0.0225 (11)*	0.204 (2)
H8BA	0.4726	0.4144	0.9508	0.027*	0.204 (2)
H8BB	0.5478	0.3612	0.7959	0.027*	0.204 (2)
C9B	0.2974 (6)	0.4397 (6)	0.8414 (6)	0.0203 (10)	0.204 (2)
H9BA	0.2106	0.5179	0.8962	0.024*	0.204 (2)
H9BB	0.2881	0.4571	0.7437	0.024*	0.204 (2)
N3	0.77232 (14)	1.20704 (14)	0.53274 (13)	0.0356 (3)	
N4	0.04293 (17)	−0.36229 (15)	1.22228 (16)	0.0481 (4)	
C1	0.77783 (15)	0.70764 (15)	0.59144 (14)	0.0310 (3)	
H1A	0.8552	0.6156	0.5652	0.037*	
C2	0.81727 (15)	0.83368 (15)	0.55783 (13)	0.0301 (3)	
H2A	0.9207	0.8263	0.5101	0.036*	
C3	0.70001 (14)	0.97147 (14)	0.59648 (12)	0.0250 (2)	
C4	0.54475 (14)	0.98285 (14)	0.66949 (13)	0.0281 (3)	
H4A	0.4671	1.0749	0.6957	0.034*	
C5	0.50763 (14)	0.85626 (14)	0.70243 (13)	0.0274 (3)	
H5A	0.4043	0.8635	0.7507	0.033*	
C6	0.62345 (14)	0.71778 (14)	0.66410 (12)	0.0248 (2)	
C7	0.58542 (16)	0.58172 (14)	0.69901 (14)	0.0302 (3)	
H7A	0.6682	0.4882	0.6713	0.036*	0.796 (2)
H7B	0.6427	0.4971	0.6419	0.036*	0.204 (2)
C10	0.18277 (17)	0.28390 (16)	1.04041 (17)	0.0379 (3)	
H10A	0.0979	0.3754	1.0711	0.045*	0.796 (2)
H10B	0.1389	0.3631	1.1042	0.045*	0.204 (2)
C11	0.15451 (14)	0.14439 (14)	1.07915 (13)	0.0269 (3)	
C12	0.00891 (15)	0.14545 (14)	1.16669 (14)	0.0309 (3)	
H12A	−0.0691	0.2345	1.2005	0.037*	
C13	−0.02034 (15)	0.01501 (14)	1.20368 (13)	0.0285 (3)	
H13A	−0.1174	0.0161	1.2625	0.034*	
C14	0.09638 (15)	−0.11766 (14)	1.15229 (13)	0.0269 (3)	
C15	0.24291 (15)	−0.12015 (15)	1.06462 (14)	0.0316 (3)	
H15A	0.3205	−0.2092	1.0303	0.038*	
C16	0.27163 (14)	0.01010 (14)	1.02930 (13)	0.0283 (3)	
H16A	0.3695	0.0086	0.9720	0.034*	
C17	0.73983 (15)	1.10277 (15)	0.56153 (13)	0.0281 (3)	
C18	0.06622 (17)	−0.25355 (16)	1.19090 (15)	0.0348 (3)	

### Atomic displacement parameters (Å^2^)

**Table:**

	*U*^11^	*U*^22^	*U*^33^	*U*^12^	*U*^13^	*U*^23^
N1A	0.0290 (7)	0.0270 (7)	0.0282 (7)	−0.0139 (5)	−0.0013 (6)	−0.0031 (5)
N2A	0.0304 (6)	0.0287 (6)	0.0298 (7)	−0.0149 (5)	−0.0010 (5)	−0.0024 (5)
C8A	0.0276 (7)	0.0234 (7)	0.0311 (8)	−0.0119 (6)	−0.0002 (6)	−0.0034 (6)
C9A	0.0340 (8)	0.0303 (8)	0.0313 (8)	−0.0176 (6)	0.0008 (6)	−0.0062 (6)
C19A	0.0537 (10)	0.0276 (7)	0.0222 (7)	−0.0273 (7)	−0.0126 (6)	0.0023 (5)
N2B	0.0304 (6)	0.0287 (6)	0.0298 (7)	−0.0149 (5)	−0.0010 (5)	−0.0024 (5)
C9B	0.024 (2)	0.016 (2)	0.021 (2)	−0.0076 (19)	−0.0053 (18)	0.0005 (18)
N3	0.0337 (6)	0.0363 (6)	0.0387 (6)	−0.0187 (5)	0.0002 (5)	−0.0055 (5)
N4	0.0457 (7)	0.0340 (7)	0.0585 (8)	−0.0165 (6)	0.0000 (6)	0.0008 (6)
C1	0.0258 (6)	0.0283 (6)	0.0341 (6)	−0.0090 (5)	0.0027 (5)	−0.0060 (5)
C2	0.0239 (5)	0.0335 (7)	0.0312 (6)	−0.0127 (5)	0.0020 (4)	−0.0046 (5)
C3	0.0253 (5)	0.0294 (6)	0.0229 (5)	−0.0134 (5)	−0.0033 (4)	−0.0031 (4)
C4	0.0235 (5)	0.0265 (6)	0.0325 (6)	−0.0096 (4)	−0.0006 (4)	−0.0051 (5)
C5	0.0225 (5)	0.0294 (6)	0.0298 (6)	−0.0116 (5)	−0.0006 (4)	−0.0036 (5)
C6	0.0252 (5)	0.0268 (6)	0.0219 (5)	−0.0112 (4)	−0.0011 (4)	−0.0029 (4)
C7	0.0304 (6)	0.0258 (6)	0.0308 (6)	−0.0108 (5)	0.0010 (5)	−0.0025 (5)
C10	0.0293 (6)	0.0273 (6)	0.0558 (9)	−0.0129 (5)	0.0018 (6)	−0.0112 (6)
C11	0.0248 (5)	0.0261 (6)	0.0312 (6)	−0.0105 (4)	−0.0043 (4)	−0.0055 (4)
C12	0.0260 (6)	0.0257 (6)	0.0360 (6)	−0.0066 (5)	0.0000 (5)	−0.0066 (5)
C13	0.0236 (5)	0.0305 (6)	0.0283 (6)	−0.0094 (5)	−0.0015 (4)	−0.0008 (5)
C14	0.0269 (6)	0.0263 (6)	0.0263 (5)	−0.0094 (5)	−0.0046 (4)	−0.0005 (4)
C15	0.0271 (6)	0.0265 (6)	0.0358 (6)	−0.0072 (5)	0.0004 (5)	−0.0057 (5)
C16	0.0232 (5)	0.0299 (6)	0.0303 (6)	−0.0100 (5)	−0.0006 (4)	−0.0054 (5)
C17	0.0256 (5)	0.0330 (6)	0.0269 (6)	−0.0134 (5)	−0.0013 (4)	−0.0054 (5)
C18	0.0326 (6)	0.0304 (7)	0.0372 (7)	−0.0113 (5)	−0.0012 (5)	−0.0005 (5)

### Geometric parameters (Å, °)

**Table:**

N1A—C7	1.2878 (18)	C2—C3	1.3947 (18)
N1A—C8A	1.4714 (19)	C2—H2A	0.9300
N2A—C10	1.2484 (18)	C3—C4	1.3979 (16)
N2A—C9A	1.455 (2)	C3—C17	1.4400 (16)
C8A—C9A	1.523 (2)	C4—C5	1.3818 (16)
C8A—C19A	1.558 (2)	C4—H4A	0.9300
C8A—H8AA	0.9800	C5—C6	1.3944 (17)
C9A—H9AA	0.9700	C5—H5A	0.9300
C9A—H9AB	0.9700	C6—C7	1.4758 (16)
C19A—H19A	0.9600	C7—H7A	0.9600
C19A—H19B	0.9600	C7—H7B	0.9600
C19A—H19C	0.9600	C10—C11	1.4658 (17)
N1B—C7	1.267 (6)	C10—H10A	0.9600
N1B—C8B	1.460 (8)	C10—H10B	0.9600
N2B—C9B	1.464 (7)	C11—C12	1.3945 (17)
N2B—C10	1.521 (6)	C11—C16	1.3975 (17)
C8B—C9B	1.546 (8)	C12—C13	1.3834 (17)
C8B—H8BA	0.9700	C12—H12A	0.9300
C8B—H8BB	0.9700	C13—C14	1.3897 (17)
C9B—H9BA	0.9700	C13—H13A	0.9300
C9B—H9BB	0.9700	C14—C15	1.3982 (17)
N3—C17	1.1484 (17)	C14—C18	1.4400 (18)
N4—C18	1.1489 (19)	C15—C16	1.3781 (17)
C1—C2	1.3883 (17)	C15—H15A	0.9300
C1—C6	1.3930 (16)	C16—H16A	0.9300
C1—H1A	0.9300		
			
C7—N1A—C8A	116.77 (13)	C1—C6—C5	119.43 (11)
C10—N2A—C9A	117.17 (13)	C1—C6—C7	119.38 (11)
N1A—C8A—C9A	107.68 (13)	C5—C6—C7	121.19 (10)
N1A—C8A—C19A	108.24 (13)	N1B—C7—N1A	24.5 (2)
C9A—C8A—C19A	111.02 (13)	N1B—C7—C6	117.3 (3)
N1A—C8A—H8AA	110.0	N1A—C7—C6	122.07 (12)
C9A—C8A—H8AA	110.0	N1B—C7—H7A	117.8
C19A—C8A—H8AA	110.0	N1A—C7—H7A	119.0
N2A—C9A—C8A	110.09 (13)	C6—C7—H7A	119.0
N2A—C9A—H9AA	109.6	N1B—C7—H7B	121.9
C8A—C9A—H9AA	109.6	N1A—C7—H7B	111.9
N2A—C9A—H9AB	109.6	C6—C7—H7B	120.8
C8A—C9A—H9AB	109.6	H7A—C7—H7B	23.9
H9AA—C9A—H9AB	108.2	N2A—C10—C11	121.33 (13)
C7—N1B—C8B	115.5 (5)	N2A—C10—N2B	31.9 (2)
C9B—N2B—C10	118.6 (4)	C11—C10—N2B	116.6 (2)
N1B—C8B—C9B	110.0 (5)	N2A—C10—H10A	119.5
N1B—C8B—H8BA	109.7	C11—C10—H10A	119.2
C9B—C8B—H8BA	109.7	N2B—C10—H10A	114.8
N1B—C8B—H8BB	109.7	N2A—C10—H10B	107.4
C9B—C8B—H8BB	109.7	C11—C10—H10B	121.7
H8BA—C8B—H8BB	108.2	N2B—C10—H10B	121.8
N2B—C9B—C8B	107.0 (4)	H10A—C10—H10B	32.0
N2B—C9B—H9BA	110.3	C12—C11—C16	119.49 (11)
C8B—C9B—H9BA	110.3	C12—C11—C10	119.68 (11)
N2B—C9B—H9BB	110.3	C16—C11—C10	120.83 (11)
C8B—C9B—H9BB	110.3	C13—C12—C11	120.51 (11)
H9BA—C9B—H9BB	108.6	C13—C12—H12A	119.7
C2—C1—C6	120.64 (11)	C11—C12—H12A	119.7
C2—C1—H1A	119.7	C12—C13—C14	119.49 (11)
C6—C1—H1A	119.7	C12—C13—H13A	120.3
C1—C2—C3	119.28 (11)	C14—C13—H13A	120.3
C1—C2—H2A	120.4	C13—C14—C15	120.52 (12)
C3—C2—H2A	120.4	C13—C14—C18	119.56 (11)
C2—C3—C4	120.53 (11)	C15—C14—C18	119.92 (11)
C2—C3—C17	119.61 (10)	C16—C15—C14	119.61 (11)
C4—C3—C17	119.86 (11)	C16—C15—H15A	120.2
C5—C4—C3	119.44 (11)	C14—C15—H15A	120.2
C5—C4—H4A	120.3	C15—C16—C11	120.37 (11)
C3—C4—H4A	120.3	C15—C16—H16A	119.8
C4—C5—C6	120.68 (11)	C11—C16—H16A	119.8
C4—C5—H5A	119.7	N3—C17—C3	179.50 (14)
C6—C5—H5A	119.7	N4—C18—C14	179.53 (16)
			
C7—N1A—C8A—C9A	134.28 (16)	C1—C6—C7—N1B	152.5 (3)
C7—N1A—C8A—C19A	−105.62 (18)	C5—C6—C7—N1B	−27.5 (4)
C10—N2A—C9A—C8A	−136.91 (16)	C1—C6—C7—N1A	−179.80 (14)
N1A—C8A—C9A—N2A	−175.76 (13)	C5—C6—C7—N1A	0.2 (2)
C19A—C8A—C9A—N2A	65.92 (17)	C9A—N2A—C10—C11	−178.46 (13)
C7—N1B—C8B—C9B	−116.7 (5)	C9A—N2A—C10—N2B	90.8 (4)
C10—N2B—C9B—C8B	105.6 (5)	C9B—N2B—C10—N2A	−83.9 (6)
N1B—C8B—C9B—N2B	−177.4 (5)	C9B—N2B—C10—C11	168.8 (4)
C6—C1—C2—C3	−0.6 (2)	N2A—C10—C11—C12	175.33 (15)
C1—C2—C3—C4	0.61 (19)	N2B—C10—C11—C12	−148.5 (3)
C1—C2—C3—C17	−179.77 (11)	N2A—C10—C11—C16	−4.8 (2)
C2—C3—C4—C5	−0.45 (19)	N2B—C10—C11—C16	31.4 (3)
C17—C3—C4—C5	179.94 (11)	C16—C11—C12—C13	−0.31 (19)
C3—C4—C5—C6	0.25 (19)	C10—C11—C12—C13	179.60 (12)
C2—C1—C6—C5	0.38 (19)	C11—C12—C13—C14	−0.35 (19)
C2—C1—C6—C7	−179.66 (11)	C12—C13—C14—C15	0.44 (19)
C4—C5—C6—C1	−0.22 (19)	C12—C13—C14—C18	179.89 (12)
C4—C5—C6—C7	179.83 (11)	C13—C14—C15—C16	0.1 (2)
C8B—N1B—C7—N1A	83.2 (8)	C18—C14—C15—C16	−179.31 (12)
C8B—N1B—C7—C6	−168.7 (4)	C14—C15—C16—C11	−0.8 (2)
C8A—N1A—C7—N1B	−97.6 (8)	C12—C11—C16—C15	0.90 (19)
C8A—N1A—C7—C6	176.59 (13)	C10—C11—C16—C15	−179.01 (12)
